# Neural mass modeling of power-line magnetic fields effects on brain activity

**DOI:** 10.3389/fncom.2013.00034

**Published:** 2013-04-11

**Authors:** J. Modolo, A. W. Thomas, A. Legros

**Affiliations:** ^1^Human Threshold Research Group, Lawson Health Research InstituteLondon, ON, Canada; ^2^Department of Medical Biophysics, Western UniversityLondon, ON, Canada; ^3^Department of Medical Imaging, Western UniversityLondon, ON, Canada; ^4^School of Kinesiology, Western UniversityLondon, ON, Canada

**Keywords:** neural mass models, power-line magnetic fields, electroencephalogram (EEG), synaptic plasticity, brain stimulation

## Abstract

Neural mass models are an appropriate framework to study brain activity, combining a high degree of biological realism while being mathematically tractable. These models have been used, with a certain success, to simulate brain electric (electroencephalography, EEG) and metabolic (functional magnetic resonance imaging, fMRI) activity. However, concrete applications of neural mass models have remained limited to date. Motivated by experimental results obtained in humans, we propose in this paper a neural mass model designed to study the interaction between power-line magnetic fields (MFs) (60 Hz in North America) and brain activity. The model includes pyramidal cells; dendrite-projecting, slow GABAergic neurons; soma-projecting, fast GABAergic neurons; and glutamatergic interneurons. A simple phenomenological model of interaction between the induced electric field and neuron membranes is also considered, along with a model of post-synaptic calcium concentration and associated changes in synaptic weights Simulated EEG signals are produced in a simple protocol, both in the absence and presence of a 60 Hz MF. These results are discussed based on results obtained previously in humans. Notably, results highlight that (1) EEG alpha (8–12 Hz) power can be modulated by weak membrane depolarizations induced by the exposure; (2) the level of input noise has a significant impact on EEG power modulation; and (3) the threshold value in MF flux density resulting in a significant effect on the EEG depends on the type of neuronal populations modulated by the MF exposure. Results obtained from the model shed new light on the effects of power-line MFs on brain activity, and will provide guidance in future human experiments. This may represent a valuable contribution to international regulation agencies setting guidelines on MF values to which the general public and workers can be exposed.

## Introduction

Since the pioneering work of Wilson and Cowan (1973) and Amari (1977), neural field models have been increasingly used, expanded and studied by a developing multidisciplinary community including: mathematicians, physicists, neuroscientists, medical imaging scientists *etcetera*. Neural field models provide a concise, yet insightful description of cortical activity. This theory has not only led to successful reproduction of numerous experimental results, but also to the prediction of a certain number of phenomena that have been observed *in vivo* (for a review of these phenomena, see Modolo et al., 2010). A popular simplification of neural field models consists in neglecting the role of space, consider neural populations present in cortical columns (such as pyramidal neurons) and to consider a connectivity matrix between the different neural masses considered (Wendling et al., 2002; Sotero and Trujillo-Barreto, 2008; Bojak et al., 2010). This approach has been used to build large-scale models of brain activity, including models simulating the electroencephalogram (EEG), or the blood oxygen level-dependent signal (BOLD) reflecting metabolic activity of brain tissue (Wendling et al., 2002; Sotero and Trujillo-Barreto, 2008; Bojak et al., 2010). Indeed, since neural mass models are tractable and make the link between local variables and observables, these models appear as an excellent comprise between biological realism and computational complexity. Therefore, here we present an application of neural mass model to a specific question that many teams over the world have been tackling for a number of years using various neuroimaging modalities: how do power-line frequency (60 Hz in North America) magnetic fields (MFs) interact with human brain activity (interaction mechanisms), and how does it translate into observable outcomes (neuroimaging data such as EEG, motor/cognitive performance)?

The effects of extremely low-frequency (categorized as being <300 Hz) MF such as power-line MF on human neurophysiology have been studied for several decades. Despite an impressive amount of experimental data *in vitro, in vivo*, and in humans, a complete understanding of the interaction mechanisms and associated effects is still to be achieved. One complication in comparing outcomes from these studies is the wide range of MF flux densities, MF exposure setups, and exposure protocols used (Crasson, 2003). First, significant work has been done has been done *in vivo* and *in vitro* in order to characterize the effects of electric fields in terms of membrane potential perturbation, excitability and neural network oscillations (Jefferys et al., 2003; Bikson et al., 2004; Deans et al., 2007; Fröhlich and McCormick, 2010; Reato et al., 2010). These studies have highlighted the role of neuronal morphology and orientation were critical in understanding the interaction with electric field, but also that membrane depolarization far below the firing threshold can influence the activity of neuronal networks. Second, among the reported effects in humans, let us mention the modulation of pain threshold (Ghione et al., 2005), effects on resting tremor (Legros and Beuter, 2005), modulations in functional brain activity as measured by BOLD (Legros et al., 2011), interference with learning in a short-term memory test (Corbacio et al., 2011). The most established interaction mechanism of MF exposure consists in induced currents, resulting from Faraday's law of induction, stating that a time-varying MF induces a time-varying electric field. This electric field will induce charge movement, creating a current. This is termed as the *induced current mechanism* (National Institute of Environmental Health Sciences of the National Institutes of Health, 1998). The induced current mechanism will be the mechanism considered in this paper. Interestingly, several studies have reported lasting effects associated with ELF MF exposure, i.e., an effect that is still detectable after cessation of the exposure. Using a specific pulsed MF, modulations of the EEG have been observed post-exposure (Cook et al., 2004, 2005). In the case of 60 Hz MF in the millitesla range (1.8 and 3 mT), modulations of the BOLD signal measured in humans during motor (finger tapping) and cognitive (mental rotation) tasks (Legros et al., 2010; Miller et al., 2010) have been found to be modulated post-exposure.

The interest for the interaction between power-line MF and human neurophysiology is twofold. First, there is a growing concern from the general public regarding the possible deleterious effects of power-line MF on human health, even if such negative effects remain to be demonstrated. Second, international regulation agencies such as ICNIRP (International Commission on Non-Ionizing Radiation Protection, http://www.icnirp.de) need results from the scientific literature to set their exposure guidelines (ICNIRP, 2010), aiming to protect the general public and workers, that are used by governments. One of the most long awaited data is the threshold in MF flux density at 60 Hz resulting in detectable effects in humans. One well-known effect of ELF MF exposure is magnetophosphenes, the perception of flickering lights in the visual field in the presence of a sufficiently strong MF. ICNIRP states that: “*Since the perception of magnetophosphenes constitutes the most reliable effect of MF exposure on human biology, this serves as a basis for the ICNIRP guidelines*” (ICNIRP, 2010). However, no data is available at 60 Hz regarding threshold values, therefore new approaches that could assist in the interpretation of existing experimental results, but also in the prediction of new results such as an estimation of threshold values that could be tested experimentally, would constitute significant advances in the field.

In order to shed light on the mechanisms involved in lasting effects of 60 Hz MF exposure on human neurophysiology, and also provide an estimate of the threshold value resulting in detectable changes in EEG caused by 60 Hz MF exposure, we present in this paper a neural mass model aiming to model brain tissue dynamics at different time scales, bridging biophysical mechanisms with changes in observables. Among the panel of brain tissue dynamics models available, the approach initiated by Jansen and Rit (1995), consisting in considering sub-populations of neurons synaptically connected; later extended and improved by Wendling et al. (2002), provides a meaningful and accurate description of cortical dynamics. Indeed, such models have been successfully applied to understand the transition between baseline EEG and epileptic activity (Wendling et al., 2002, 2005; Molaee-Ardekani et al., 2010). First, we present an extension of the neural mass model developed by Sotero and Trujillo-Barreto (2008), by including a population of fast GABAergic neurons as suggested by Wendling et al. (2002). Second, we include a simple biophysical model of interaction between the 60 Hz MF and neuronal activity, along with a model of synaptic plasticity changes related to post-synaptic calcium concentration levels (Shouval et al., 2002a,b). Third, we use this model to investigate *in silico* the effects of 60 Hz MF on cortical dynamics, notably to evaluate the threshold in MF flux density resulting in detectable changes in variables of interest. The role of synaptic input noise, neuronal populations modulated by the exposure, and synaptic plasticity are also explored. Finally, we discuss future directions of research using this modeling approach.

## Materials and methods

### Neural mass model

In order to develop a biologically grounded model to study the effect of 60 Hz MF on neuronal activity, we have extend the thalamo-cortical model proposed by Sotero and Trujillo-Barreto (2008) by including a population of soma-projecting, fast inhibitory γ-amino-butyric acid (GABA) interneurons to extend the possible dynamical repertoire of the model [as shown by Wendling et al. (2002)]. The proposed modification of the block diagram proposed by Sotero and Trujillo-Barreto (2008) used to describe the thalamo-cortical model is the following:

Model equations are obtained by using the fact that the synaptic response function (Green's function) for a type of synapse *i* (e.g., glutamatergic) writes as *V*_*i*_(*t*) = *A*_*i*_.*a*_*i*_.*t*. exp(−*a*_*i*_.*t*), where *A*_*i*_ is the response amplitude and *a*_*i*_ the response time constant. Considering the temporal operator $\hat{L}=\frac{d^{2}}{dt^{2}}+2a_{i}\frac{d}{dt}+a_{i}^{2}$, and using the fact that the synaptic response is a Greens' function for the temporal operator, we can use $\hat{L}V_{i}(t)=\delta (t)$ to write the following neural mass equation:

(1) $$\frac{d^{2}}{dt^{2}}V_{i}(t)+2.a.\frac{d}{dt}V_{i}(t)+a^{2}.V_{i}(t)=A.a.\upsilon_{i}(t)$$

where υ_*i*_(*t*)is the incoming firing rate. It is often practical to write Equation (1) under the form of a system of two first-order differential equations:

(2) $$\begin{array}{l} \frac{d}{dt}V_{i}(t)=y_{i}(t)\\ \frac{d}{dt}y_{i}(t)=A.a.\upsilon_{i}(t)-2.a.\frac{d}{dt}y_{i}(t)+a^{2}.V_{i}(t) \end{array}$$

**Figure 1:**
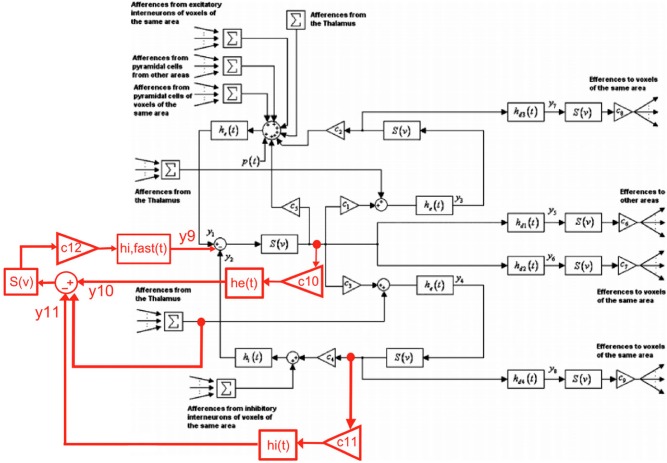
**Proposed extension of the Sotero et al. model of cortical dynamics (figure modified from (Sotero and Trujillo-Barreto, 2008); with permission).** The inclusion of the new population of fast GABAergic neurons and its connectivity with other neuronal populations is highlighted in red.

Using this principle for each block of EPSP/IPSP presented in Figure 1, it is possible to formulate our extended thalamo-cortical model as a system of 22 differential equations (6 new equations corresponding to the new population of fast inhibitory interneurons, its feedback loop with pyramidal neurons, and its inhibitory input from slow inhibitory interneurons) presented below:

(3) $$\begin{array}{l} {\dot{y}}_{1}^{nj}(t)=y_{12}^{nj}(t)\\ {\dot{y}}_{2}^{nj}(t)=y_{13}^{nj}(t)\\ {\dot{y}}_{3}^{nj}(t)=y_{14}^{nj}(t)\\ {\dot{y}}_{4}^{nj}(t)=y_{15}^{nj}(t)\\ {\dot{y}}_{5}^{nj}(t)=y_{16}^{nj}(t)\\ {\dot{y}}_{6}^{nj}(t)=y_{17}^{nj}(t)\\ {\dot{y}}_{7}^{nj}(t)=y_{18}^{nj}(t)\\ {\dot{y}}_{8}^{nj}(t)=y_{19}^{nj}(t)\\ {\dot{y}}_{9}^{nj}(t)=y_{20}^{nj}(t)\\ {\dot{y}}_{10}^{nj}(t)=y_{21}^{nj}(t)\\ {\dot{y}}_{11}^{nj}(t)=y_{22}^{nj}(t)\\ {\dot{y}}_{12}^{nj}(t)=A.a.\{c_{5}.S\left[y_{1}^{nj}(t)-y_{2}^{nj}(t)-y_{9}^{nj}(t)\right]\\ \;+\;c_{2}.S\left[y_{3}^{nj}(t)\right]+K^{th,n}c_{3}^{t}.S\left[x_{4}(t)\right]\}\\ \;+A.a.\{\sum_{\begin{array}{l} m=1\\ m\neq j \end{array}}^{M_{n}}\left(k_{e1}^{mj}.c_{7}.S\left[y_{6}^{mn}(t)\right]+k_{e2}^{mj}.c_{8}.S\left[y_{7}^{mn}(t)\right]\right)\\ \;+\sum_{\begin{array}{l} i=1\\ i\neq n \end{array}}^{N}K^{i,n}\sum_{m=1}^{M_{i}}c_{6}.S\left[y_{5}^{im}(t)\right]\}\\ \;-2.a.y_{12}^{nj}(t)-a^{2}y_{1}^{nj}(t)+A.a.p^{nj}(t)\\ {\dot{y}}_{13}^{nj}(t)=B.b.\left\{c_{4}.S\left[y_{4}^{nj}(t)\right]+\sum_{\begin{array}{l} \begin{array}{l} m=1 \end{array}\\ m\neq j \end{array}}^{M_{n}}\left(k_{i}^{mj}.c_{9}.S\left[y_{8}^{mn}(t)\right]\right)\right\}\\ \;-2.b.y_{13}^{nj}(t)-b^{2}y_{2}^{nj}(t)\\ {\dot{y}}_{14}^{nj}(t)=A.a.\left\{c_{1}.S\left[y_{1}^{nj}(t)-y_{2}^{nj}(t)-y_{9}^{nj}(t)\right]+K^{th,n}.c_{4}^{t}S\left[x_{5}(t)\right]\right\}\\ \;-2.a.y_{14}^{nj}(t)-a^{2}y_{3}^{nj}(t)\\ {\dot{y}}_{15}^{nj}(t)=A.a.\left\{c_{3}.S\left[y_{1}^{nj}(t)-y_{2}^{nj}(t)-y_{9}^{nj}(t)\right]+K^{th,n}.c_{5}^{t}S\left[x_{6}(t)\right]\right\}\\ \;-2.a.y_{15}^{nj}(t)-a^{2}y_{4}^{nj}(t)\\ {\dot{y}}_{16}^{nj}(t)=A.a_{d1}.\left\{S\left[y_{1}^{nj}(t)-y_{2}^{nj}(t)-y_{9}^{nj}(t)\right]\right\}\\ \;-2.a_{d1}.y_{16}^{nj}(t)-a_{d1}^{2}y_{5}^{nj}(t)\\ {\dot{y}}_{17}^{nj}(t)=A.a_{d2}.\left\{S\left[y_{1}^{nj}(t)-y_{2}^{nj}(t)-y_{9}^{nj}(t)\right]\right\}\\ \;-2.a_{d2}.y_{17}^{nj}(t)-a_{d2}^{2}y_{6}^{nj}(t)\\ {\dot{y}}_{18}^{nj}(t)=A.a_{d3}.\left\{S\left[y_{3}^{nj}(t)\right]\right\}-2.a_{d3}.y_{18}^{nj}(t)-a_{d3}^{2}y_{7}^{nj}(t)\\ {\dot{y}}_{19}^{nj}(t)=B.b_{d4}.\left\{S\left[y_{4}^{nj}(t)\right]\right\}-2.b_{d4}.y_{19}^{nj}(t)-b_{d4}^{2}y_{8}^{nj}(t)\\ {\dot{y}}_{20}^{nj}(t)=G.g.\left\{c_{12}.S\left[y_{10}^{nj}(t)-y_{11}^{nj}(t)\right]+K^{th,n}.c_{5}^{t}S\left[x_{6}(t)\right]\right\}\\ \;-2.g.y_{20}^{nj}(t)-g^{2}y_{9}^{nj}(t)\\ {\dot{y}}_{21}^{nj}(t)=A.a.\left\{c_{10}.S\left[y_{1}^{nj}(t)-y_{2}^{nj}(t)-y_{9}^{nj}(t)\right]\right\}\\ \;-2.a.y_{21}^{nj}(t)-a^{2}y_{10}^{nj}(t)\\ {\dot{y}}_{22}^{nj}(t)=B.b.\left\{c_{11}.S\left[y_{4}^{nj}(t)\right]\right\}-2.b.y_{22}^{nj}(t)-b^{2}y_{11}^{nj}(t) \end{array}$$

The equations describing the activity of the thalamus, composed of a population of thalamocortical cells and a population of reticular cells are [modified from Sotero and Trujillo-Barreto (2008)]:

(4) $$\begin{array}{l} {\dot{x}}_{1}(t)=x_{7}(t)\\ {\dot{x}}_{2}(t)=x_{8}(t)\\ {\dot{x}}_{3}(t)=x_{9}(t)\\ {\dot{x}}_{4}(t)=x_{10}(t)\\ {\dot{x}}_{5}(t)=x_{11}(t)\\ {\dot{x}}_{6}(t)=x_{12}(t)\\ {\dot{x}}_{7}(t)=A_{t}.a_{t}.\left\{\sum_{i=1}^{N}K^{th,i}\sum_{m=1}^{M_{i}}c_{6}.S\left[y_{5}^{im}(t)+p_{th}(t)\right]\right\}\\ \;-2.a_{t}.x_{7}(t)-a_{t}^{2}x_{1}(t)\\ {\dot{x}}_{8}(t)=B_{t}.b_{t}.c_{2t}.S\left[c_{1t}x_{3}(t)\right]-2.b_{t}.x_{8}(t)-b_{t}^{2}x_{2}(t)\\ {\dot{x}}_{9}(t)=A_{t}.a_{t}.S\left[x_{1}(t)-x_{2}(t)\right]-2.a_{t}.x_{9}(t)-a_{t}^{2}x_{3}(t)\\ {\dot{x}}_{10}(t)=A_{t}.a_{d1t}.S\left[x_{1}(t)-x_{2}(t)\right]-2.a_{d1t}.x_{10}(t)-a_{d1t}^{2}x_{4}(t)\\ {\dot{x}}_{11}(t)=A_{t}.a_{d2t}.S\left[x_{1}(t)-x_{2}(t)\right]-2.a_{d2t}.x_{11}(t)-a_{d2t}^{2}x_{5}(t)\\ {\dot{x}}_{12}(t)=A_{t}.a_{d3t}.S\left[x_{1}(t)-x_{2}(t)\right]-2.a_{d3t}.x_{12}(t)-a_{d3t}^{2}x_{6}(t) \end{array}$$

The physical meaning and values of model parameters are detailed in Table A1 (Appendix section). For more details, the reader can refer to Sotero and Trujillo-Barreto (2008). Overall, the model is composed of 22 differential equations describing cortical dynamics of four different neuronal populations (pyramidal neurons, glutamatergic interneurons, fast/slow GABAergic neurons, and 12 differential equations defining thalamic activity). Therefore, this set of 34 differential equations describes the thalamocortical activity including time delays between cortical areas, connectivity parameters, synaptic responses derived from neurophysiology, and a biologically plausible (even if it is obviously simplified) circuitry between the neuronal populations considered. Table A1 provided in Appendix summarizes the parameters used in the model, with new parameters added due to the population of fast inhibitory interneurons that have been highlighted.

### Model of interaction between 60 HZ exposure and neuron membranes

In order to model the interaction between the electric field induced by 60 Hz MF exposure and neural tissue, we have used the « λ ·*E* » model in order to simulate the modulation of neuron membrane polarization (Molaee-Ardekani et al., 2013). In this model, the membrane depolarization *dV* in the presence of an electric field **E** is a function of a constant **λ** termed “polarization length” (Radman et al., 2009). More precisely, the membrane depolarization is expressed as *dV* = **λ**·***E***, where **λ** is a vector oriented along the neuron fibre, and ***E*** is the electric field vector. This expression is valid for a static electric field. In the case of a time-varying electric field (such as the electric field induced by 60 Hz MF), a frequency-dependent term needs to be included (Gianni et al., 2006), resulting in:

(5) $$dV=\frac{\lambda \cdot E}{\sqrt{1+\omega^{2}\tau^{2}}}$$

where ω = *2*π*f, f* being the frequency (in our case, *f* = 60 Hz), and τ is the polarization time constant. In order to use Equation (5), describing membrane depolarization induced by the induced electric field, at the level of a neuronal population, we made the following assumptions: (1) the induced 60 Hz electric field is homogeneous in space at the level of the neural mass (i.e., the MF flux density is constant at each point of the cortical column); (2) the MF-induced membrane depolarization is applied to pyramidal neurons only because of their large size compared to other types of neurons in the human cortex; (3) pyramidal neurons in a given neural mass all have the same spatial orientation. Taken together, assumptions (1) and (3) result in identical *dV* values for all pyramidal neurons in a neural mass at a given time. These assumptions lead to the use Equation (3) in the context of a neural mass model.

This was achieved by modifying the expression of the total post-synaptic potential at the level of pyramidal neurons:

(6) $$y_{1}(t)-y_{2}(t)-y_{9}(t)\to y_{1}(t)-y_{2}(t)-y_{9}(t)+dV(t)$$

As it is commonly calculated in neural mass model, the EEG signal was computed as the summation of excitatory and inhibitory post-synaptic potential at the level of pyramidal neurons:

(7) $$EEG(t)=y_{1}(t)-y_{2}(t)-y_{9}(t)+dV(t)$$

Let us mention that, even if the MF-induced depolarization is included in the model as a simple additive perturbation, it has the potential to induce non-linear effects. Indeed, the effective potential at the level of pyramidal neurons (7) is used as an input for other neuronal populations, and is transformed from a potential to a firing rate using a sigmoid function, which is fundamentally non-linear. In the “Results” section, we have used arbitrary values for the field-induced membrane depolarization *dV*, guided by preliminary simulation results. Based on *dV* values resulting in significant changes in the EEG alpha power with or without synaptic plasticity in the model, we will provide an estimate of the corresponding level of 60 Hz MF flux density. This will provide us with an order of magnitude of the 60 Hz MF flux density threshold value that should result in effects detectable experimentally in humans.

In our simulations, we focused specifically on the EEG alpha rhythm (8–12 Hz). The reason of this choice is twofold. First, as mentioned in the Introduction, there is converging evidence that extremely low-frequency MF in the millitesla range, such as 60 Hz MF, can induce EEG alpha activity modulation. Second, the model we have developed is basically an extension of the Jansen and Rit model, designed to model EEG alpha activity. It is possible to reproduce other types of EEG rhythms (e.g., beta −13 to 30 Hz), for example by introducing heterogeneity in the time constant of neural populations over different neural masses (Wendling et al., 2002), which exceeds the scope of this paper.

### Biophysical model of synaptic plasticity

In order to investigate the hypothesis that 60 Hz MF exposure might modulate with human neurophysiology by modulating synaptic plasticity, we have implemented a simplified model of synaptic plasticity, based on the biophysical model developed by Shouval et al. (2002a,b). It is now well accepted that the mechanisms of long-term synaptic potentiation and depression (LTP/LTD, respectively) involve changes in post-synaptic calcium concentration and the trafficking of α-amino-3-hydroxy-5-methyl-4-isoxazole-propionic acid (AMPA) glutamate receptors between the intracellular medium and the synapse site. Depending on the calcium concentration, AMPA receptors can either insert into the membrane at the level of the synaptic cleft, or undergo an endocytosis, which is termed *receptor trafficking* (Collingridge et al., 2004). An increase in the number of AMPA receptors at the synaptic level will increase the number of glutamate molecules that can bind on post-synaptic membranes, thereby increasing membrane depolarization during a synaptic event. Consequently, the number of post-synaptic AMPA receptors is directly proportional to the synaptic weight. The model proposed by Shouval et al. (2002a,b) has been a significant progress in the modeling of the biophysical processes at play during LTP/LTD. This model is based on the “calcium control hypothesis,” according to which the level of post-synaptic calcium is the main factor regulating the exocytosis/endocytosis rate of AMPA receptors, and therefore the dynamics of synaptic plasticity changes.

We have adapted the model by Shouval et al. (2002a,b) to our neural mass model, and despite some simplifications with respects to the original model; our synaptic plasticity model captures some of its essential features. Based on the experimental literature on 60 Hz MF exposure effects on the EEG alpha rhythm, we assume that (1) no qualitative changes of EEG dynamics will occur due to 60 Hz MF exposure, changes will be purely quantitative (i.e., EEG alpha rhythm amplitude/spectral power changes, but no qualitative change in dynamical regime such as a transition toward high-amplitude, low-frequency spiking); (2) the coupling between the synaptic plasticity model and the 60 Hz MF is *via* the equation linking the EEG with the post-synaptic calcium concentration, occurring on long timescales (depending on the opening of N-methyl-D-aspartate (NMDA) glutamate receptors, not represented in the model). Therefore, the model offers the possibility to test the hypothesis that 60 Hz MF exposure can modulate synaptic plasticity by interfering with the calcium fluxes at the level of synapses. However, it does not take into account possible effects of 60 Hz MF exposure on spike timing (see the “Discussion” section).

Let us consider the average calcium post-synaptic concentration in a neural mass. The model proposed by Shouval et al. (2002a,b) links the calcium current at the level of NMDA receptors with the calcium concentration, and finally to a differential equation describing the dynamics of synaptic weight change as a function of two different calcium-dependent functions. The time constant of calcium concentration dynamics is long (on the order of minutes), and the calcium concentration increases with the membrane potential. Therefore, it appears reasonable to approximate calcium dynamics by a low-pass filtering of the mean potential of a given neural mass:

(8) $$\tau_{{\text{Ca}}^{2+}}\frac{d}{dt}[{\text{Ca}}^{2+}]+[{\text{Ca}}^{2+}]=\gamma (y_{1}-y_{2}-y_{9})$$

where *y*_1_ − *y*_2_ − *y*_9_ is the “EEG” signal at the level of a neural mass (e.g., summation of post-synaptic potentials at the level of pyramidal neurons as defined previously). Once the post-synaptic dendritic calcium concentration is obtained, it is possible to evaluate the calcium-dependent functions η and Ω present in the Shouval et al. (2002a,b) model, used to express the dynamics of the synaptic weight *c*_*i*_ (*i* denoting the type of synapse in the neural mass, e.g., afferent glutamatergic synapses on pyramidal neurons) at the level of a given neural mass:

(9) $$\frac{dc_{i}(t)}{dt}=\eta (t)\cdot [\Omega_{Ca^{2+}}(t)-c_{i}(t)]$$

**Figure A1:**
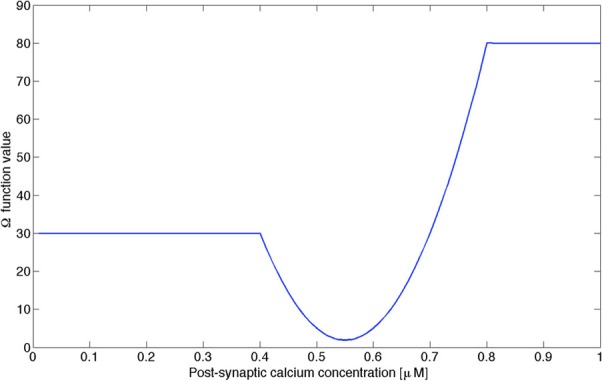
**Function Ω used in our model as a piecewise-linear approximation of the function used by Shouval et al. (2002b), preserving the main properties of this function, i.e., the level of post-synaptic calcium concentration regulating LTP and LTD processes**.

The function Ω was approximated by a combination of piecewise-linear and quadratic functions (see Appendix, Figure A1 for details) similar to the function proposed in Shouval et al. (2002b). The function Ω used in our model differs quantitatively from the one proposed in Shouval et al. (2002a,b), since the authors were linking with this function the level of post-synaptic calcium concentration with the relative change in synaptic weight, where we directly link the post-synaptic calcium concentration with the synaptic weight itself. Nevertheless, the Ω used in this paper captures the most important qualitative properties proposed by Shouval et al. (2002a,b). In our simulations, we have assumed that was η (*t*) a constant, [*Ca*^2+^(*t*)] being bounded between 0 and 1 μM. We assumed that the synapses modulated were the synapses terminating on pyramidal neurons, pooled in the constant c_5_, becoming the variable *c*_5_(*t*) in our model. Numerical implementation for the neural mass model was performed using Matlab 2010 (The Mathworks, USA) on a quad-core Apple iMac (2.66 GHz/CPU) with 8 GB of RAM. The simulation of a neural mass using the complete model during 2 h with a time step of *dt* = 1 ms took typically 8 min.

### 60 Hz MF exposure protocol

In order to study the effects of 60 Hz MF on the simulated EEG, we used the following protocol: the neural mass was simulated during 2 h overall with a 1 ms resolution, which was decomposed as (1) 30 min without 60 Hz MF exposure (termed “sham,” of sufficient duration to reach a steady state); (2) 60 min with 60 Hz MF exposure (sufficient to reach the new steady state); and (3) 30 min without 60 Hz MF exposure. Previous research in our team using fMRI to image the functional changes in brain activity due to 60 Hz MF exposure involved comparable durations (notably, a 60 min exposure period and fMRI acquisitions performed before and after, see Legros et al., 2010). Simulations were performed both (1) using the synaptic plasticity model, and (2) using a fixed synaptic weight value taken as the steady state value when synaptic plasticity was taken into account. By doing so, we aimed at decomposing the respective contribution of the 60 Hz sinusoidal perturbation in membrane potential one the one hand, and of possible calcium-related synaptic plasticity modulations on the other hand.

## Results

### Effect of 60 Hz MF on the EEG in the model

**Figure 2:**
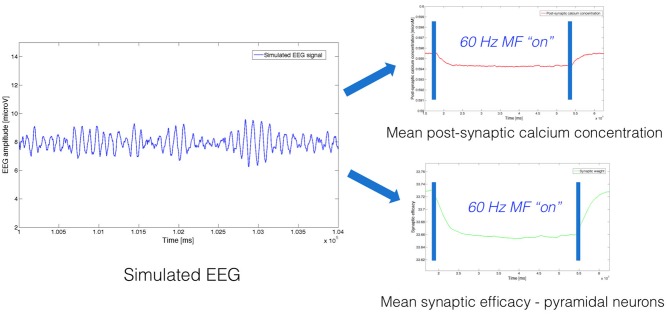
**Example of neurophysiological signals (EEG, post-synaptic calcium concentration, synaptic weight) simulated using the model, both without and with exposure to a 60 Hz MF**.

As an example, we present in Figure 2 an example of simulated EEG data, and associated mean post-synaptic calcium concentration and synaptic weight obtained by solving Equations (3, 4, 8, 9).

**Figure 3:**
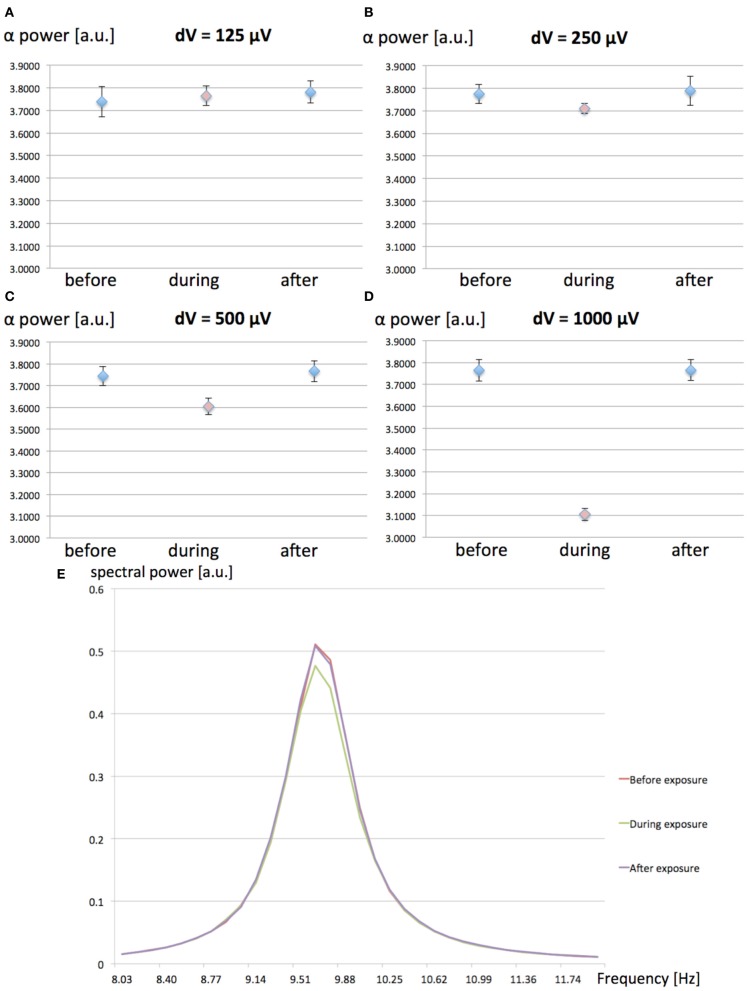
**(A–D):** Spectral power in the EEG alpha (8–12 Hz) band as a function of the MF-induced membrane polarization *dV*; before (blue), during (red) and after (blue) the 1-h 60 Hz MF exposure period. **(A)** *dV* = 125 μV; **(B)**, *dV* = 250 μV; **(C)** *dV* = 500 μV; **(D)** *dV* = 1000 μV. A decrease in EEG alpha power is observed as the value of *dV* (proportional to the MF flux density) increases. **(E)** Example of average power spectrum before, during and after exposure to the 60 Hz MF, for *dV* = 500 μV.

We have investigated the effect of increasing values (125, 250, 500, and 1000 μV) for the MF-induced membrane depolarization on the EEG alpha power. EEG alpha spectral power was computed before, during and after the 1-h 60 Hz MF exposure. 10 runs of 7200 s were performed for each tested value of *dV*. The averaged EEG alpha power for each condition (before, during, and after exposure) is presented in Figure 3.

From the results presented in Figure 3, it appears that increasing values of *dV* gradually decreases EEG alpha power during exposure. In order to test the significance of the amplitude of *dV* on the EEG alpha power during 60 Hz MF exposure, we conducted a statistical analysis of the results. We performed a 4 × 3 × 2 ANOVA for repeated measures (SPSS 21, IBM, USA), respectively testing for the effects of “*dV*” (125, 250, 500, and 1000 μV), “time” (before, during and after), and “plasticity” (with/without synaptic plasticity). The standard *p*-value of 0.05 (Greenhouse-Geisser) was chosen as the threshold for significance, and *p*-values were corrected for multiple comparisons. The statistical results reveal a significant decrease of the EEG alpha power for *dV* = 500 μV as compared to the other values of *dV* (*p* < 0.001). This indicates that the threshold for a significant decrease of EEG alpha power due to 60 Hz MF exposure lies between induced membrane depolarization values of 250–500 μV. In the next section, we attempt to link the membrane depolarization values to the corresponding MF flux density at 60 Hz.

**Figure 4:**
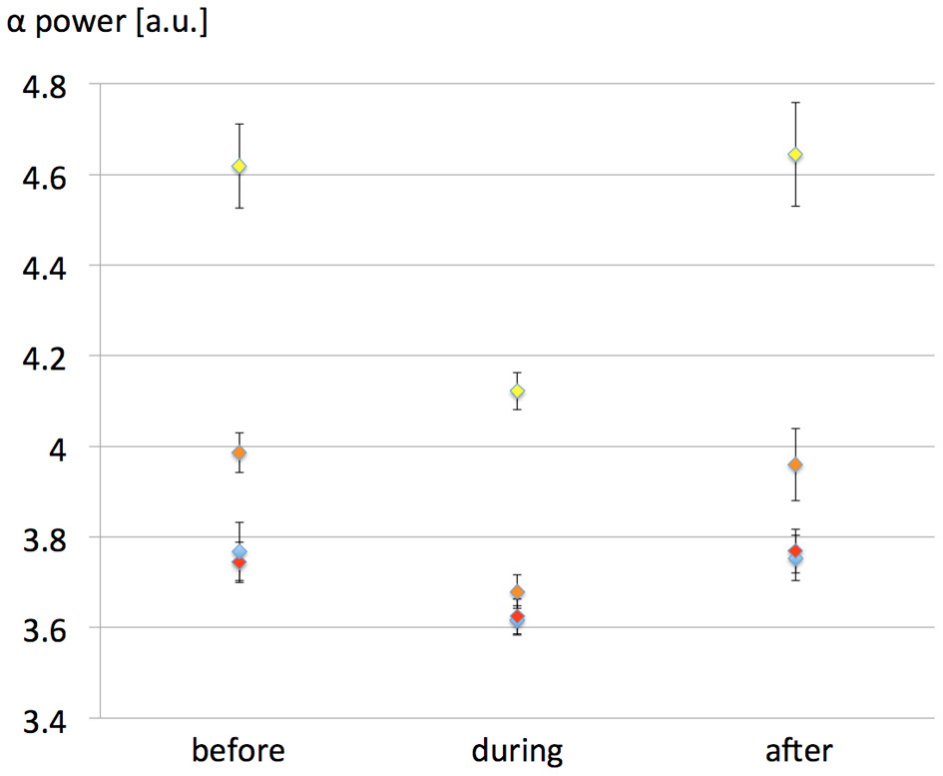
**Effect of the input noise level variance on EEG alpha power modulation caused by the 60 Hz MF exposure in the presence of the simplified synaptic plasticity model.** Noise variance σ was varied as follows: yellow, 120 spikes/s; orange: 150 spikes/s; red, 180 spikes/s; blue: 210 spikes/s. The value of *dV* was fixed to 500 μV in all simulations. The EEG alpha power is presented for each noise variance value before, during and after 60 Hz MF exposure. The impact of the 60 Hz MF exposure on EEG alpha power decreases with increased input noise amplitude, likely since the weak 60 Hz membrane potential perturbation becomes “buried” in noise.

Due to the possibility that the weak 60 Hz membrane depolarization can be seen as an additive noise, we have tested the influence of the input noise level [*p*(*t*) in the model, see Table A1 of the Appendix] variance on EEG alpha power modulation due to the exposure. Since the dynamics of the model itself depend critically on the input noise level, we have indeed investigated the possibility that the 60 Hz MF exposure has an effect of variable amplitude depending on input noise. The interest is that model predictions could be tested experimentally (e.g., in an experimental setting where different levels of visual input would be tested). Therefore, the objective was not to study the influence of the noise level on the model dynamics, but rather how the effects of the 60 Hz MF on model dynamics are dependent on the noise level. Four different values of noise variance were tested (σ = 120, 150, 180, and 210 spikes/s) for the same maximal *dV* value of 0.5 mV. 10 runs of 7200 s following the same protocol than previously were run for each noise level value (40 runs total). The influence of the input noise level on EEG alpha power modulation by the 60 Hz MF is presented in Figure 4.

The results presented in Figure 4 highlight the importance of the input noise level of the model. If the input *p*(*t*), representing external noisy input to the neural mass, is too high; then the effect induced by the 60 Hz MF on EEG alpha power modulation decreases. This has an immediate consequence on threshold values of MF flux density resulting in detectable effects in brain activity: the MF flux density needed to elicit a response in brain tissue will be lower in the presence of a low level of noise. Interestingly, there is experimental evidence that the visual input can play a role on the effects of MF exposure in humans, with an higher effect when the eyes are closed (Legros et al., 2011). EEG alpha oscillations increase dramatically eyes closed, and decrease in the presence of a visual input, that increases the input noise to the occipital cortex. Therefore, even if there is a considerable gap between the model and human data, it is tempting to make a parallel between smaller effects of 60 Hz MF in the model with high levels of noise, and smaller effects of 60 Hz MF exposure eyes open with an increased input noise level. One advantage of using our neural mass over interpreting experimental results is the possibility to point at precise mechanisms by which the observed decrease in EEG alpha activity occurs due to the 60 Hz MF exposure. From a physiological point of view, it is relevant to investigate which neuronal pathways are mainly modulated by the exposure. In the model, we observe an immediate decrease in the activity of the loop between pyramidal neurons and slow GABAergic neurons, likely increasing the effect of excitatory input. To complement this observation, it is relevant to note that, using a bifurcation theory analysis of the Jansen and Rit model (the core of our model), Grimbert and Faugeras (2006) have shown that increasing the input noise level at the level of pyramidal neurons in the alpha oscillations regime (corresponding to a Hopf bifurcation) had the effect to decrease the amplitude of alpha oscillations. Therefore, the 60 Hz MF stimulus used in our model seems to have a similar effect than an additive, positive constant membrane depolarization on pyramidal neurons. This results physiologically speaking from an efficiency decrease of the slow inhibitory GABAergic feedback at the pyramidal neurons level. In terms of dynamical systems theory, this seems to be the natural result of increased input level in a specific dynamical system on a Hopf cycle.

**Figure 5:**
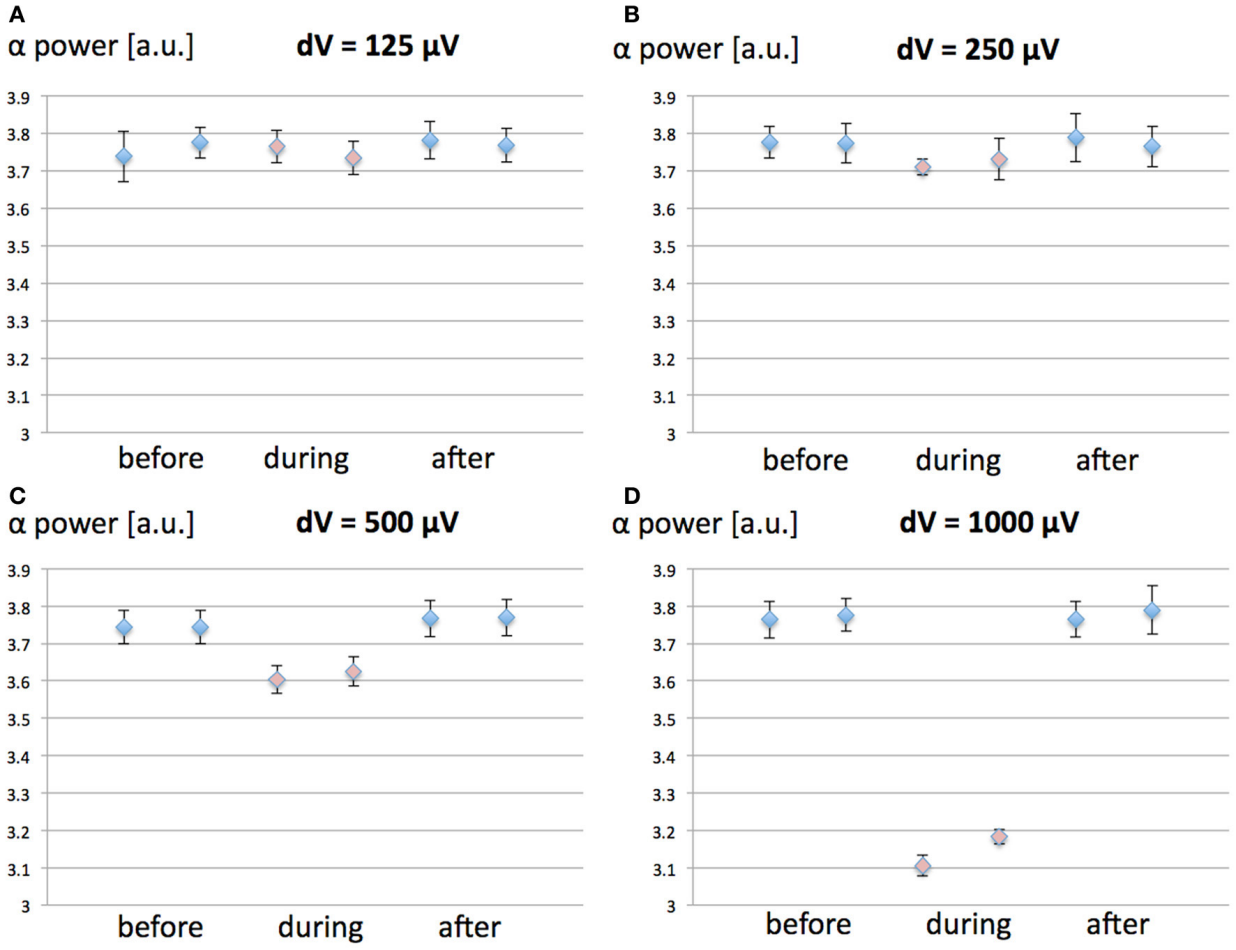
**Effect of synaptic plasticity on EEG alpha power modulation by 60 Hz MF exposure compared to the case where synaptic plasticity is not taken into account.** The conditions are Before, During, and After 60 Hz MF exposure, with (1, 3, 5) and without (2, 4, 6) synaptic plasticity. **(A)** dV = 125 μV; **(B)** dV = 250 μV; **(C)** dV = 500 μV; **(D)** dV = 1000 μV.

In order to distinguish between the contribution of the MF-induced membrane polarization on the one hand, and changes in synaptic plasticity on the other hand, we ran the same simulations than previously for four different values of *dV* (125, 250, 500, and 1000 μV), with a constant value for the synaptic weight *c*_5_. The objective was to identify if synaptic plasticity was affecting the direction (increase/decrease of EEG alpha spectral power) or amplitude of the effects. In the following, the value *c*_5_ of was chosen as the steady-state value in the case where synaptic plasticity was considered. 10 simulations of 7200 s were ran for each value of *dV*. The results are presented in Figure 5.

From the results in Figure 5, it appears that the modulation of post-synaptic calcium concentration and corresponding changes in synaptic weight plays a minimal role in EEG alpha power modulation due to the 60 Hz MF exposure, and does not impact qualitatively the result (the direction of the effects is the same, and the amplitude of the effects is minimally affected). Indeed, the results from the ANOVA shows no significant interaction effect between synaptic plasticity and *dV* values (*p* = 0.253). This indicates that the presence of the synaptic plasticity mechanisms included in the model does not significantly change the effect of the membrane depolarization. It seems however to induce a non-significant increase the amplitude of the 60 Hz MF exposure effect. Different choices for the function linking the post-synaptic calcium concentration level with the updated synaptic weight Ω lead to similar results (not shown). Therefore, it seems that, if a modulation of synaptic plasticity explains lasting effects of 60 Hz MF exposure, it does not occur primarily by the modulation of post-synaptic calcium currents. However, it is still plausible that receptor trafficking and synaptic plasticity could be impacted by a perturbation of spike timing due to the 60 Hz MF exposure, a mechanism not included in the present model, which we discuss later.

**Figure 6:**
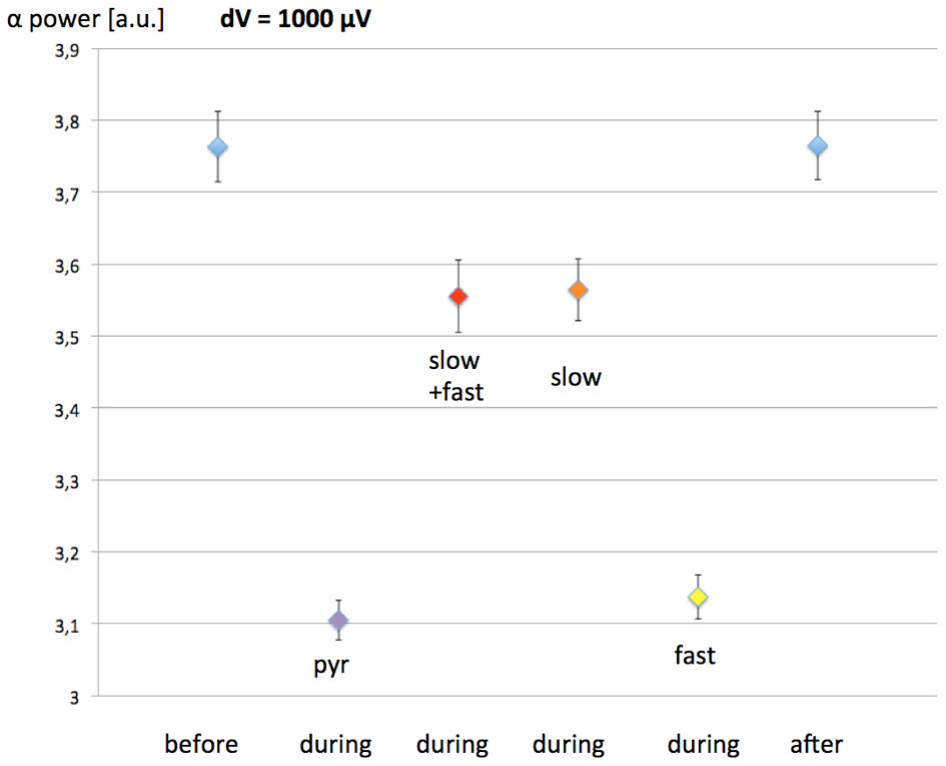
**Effect of the 60 Hz MF exposure on EEG alpha power depending on which populations of neurons are modulated by the induced electric field.** The exposure protocol is the same than previously (1-h exposure, with 30 min before and after without exposure). The maximal value of the MF-induced membrane depolarization was *dV* = 1 mV. Pyramidal neurons are considered to be modulated in each scenario. Pyr, pyramidal neurons only; slow+fast, pyramidal neurons, slow and fast GABAergic interneurons; slow, pyramidal neurons, slow GABAergic neurons; fast, pyramidal neurons, fast GABAergic neurons.

In the model develop by Molaee-Ardekani et al. (2013), investigating the effects of transcranial direct current stimulation (tDCS), the neurons being modulated by the induced field were pyramidal neurons and inhibitory interneurons. Since the simulated EEG could result in different outcomes due to the exposure depending on the neuronal populations simulated, possibly assisting in discriminating between different interaction mechanisms; we have also simulated the EEG in the case of a 60 Hz MF exposure modulating the activity of different populations of inhibitory interneurons. To do so, in a similar fashion to Equation (7) describing the membrane depolarization of pyramidal neurons, we simulated different scenarios: (1) slow and fast GABAergic interneurons are involved, (2) slow GABAergic interneurons are involved, and (3) fast inhibitory neurons are involved. Depending on the scenario, we also added the variable *dV*(*t*) to *y*_4_(*t*) (slow GABAergic interneurons), or to *y*_10_(*t*)-*y*_11_(*t*) (fast GABAergic interneurons), and to both of these quantities for scenario (1). We have simulated the EEG for a similar protocol than previously (30 min without exposure, 1 h of exposure, 30 min without exposure), with a maximal value of *dV* = 1000 μV. The resulting EEG alpha power before, during and after exposure for each of these scenarios is presented in Figure 6.

The results presented in Figure 6 show that, if the fast GABAergic interneurons are modulated by the 60 Hz MF in addition to pyramidal neurons, the difference in the EEG is minimal. However, if the slow GABAergic interneurons are modulated, the decrease in EEG alpha power is much smaller, dropping from 17% (pyramidal neurons only) to 5%. Therefore, if the slow GABAergic interneurons are also modulated by the induced electric field due to the 60 Hz MF exposure, the threshold leading to a systematic EEG alpha power modulation will be higher. From the interpretation on the decrease in EEG alpha power when the pyramidal neurons alone are stimulated, where a decrease in the efficiency of the loop between pyramidal neurons and slow inhibitory interneurons is observed, we can speculate that the effect of modulating the slow GABAergic interneurons in addition to pyramidal neurons has an opposite effect of increasing the activity of this loop. Consequently, if slow GABAergic interneurons are modulated by the exposure to the 60 Hz MF exposure, the model suggests that it would result in a compensation of the modulation of pyramidal neurons' activity alone, thereby increasing the threshold in MF flux density leading to a systematic decrease in alpha. In other words, the modulation of pyramidal neurons and slow GABAergic interneurons activity would have competing effects regarding the decrease in EEG alpha power.

### Threshold of 60 Hz MF flux density resulting in detectable effects

**Figure 7:**
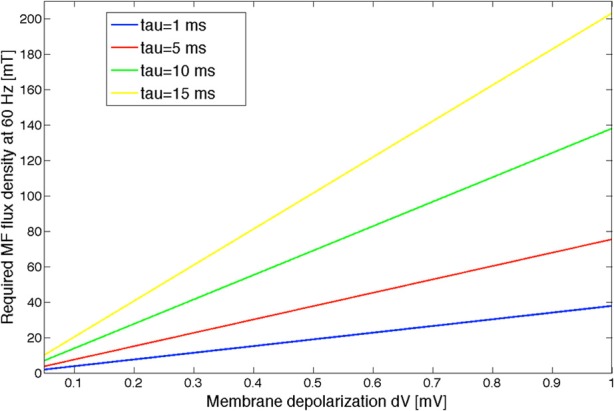
**MF flux density curves as a function of the MF-induced membrane depolarization *dV*, computed for different values of the polarization time constant τ.** The input noise variance to the model was taken as 180 spikes/s. Different noise variance values will result in different MF threshold curves.

Based on the expression of the electric field induced by a time varying MF at the level of a sphere of radius *R* (approximating the brain in that scenario), we can obtain an estimate of the corresponding MF flux density at 60 Hz. Let us approximate the head as a sphere of radius *R*, and let us write the 60 Hz MF as *B*(*t*) = *B*_0_ sin (ω*t* + ϕ). From Maxwell-Faraday's law of magnetic induction, the induced electric field expresses as $E=\frac{R}{2}\frac{dB}{dt}=\pi RfB_{0}$. By using this expression in Equation (5), we obtain $B=\frac{dV{\left(1+\omega^{2}\tau^{2}\right)}^{1/2}}{\lambda \pi Rf}$, linking the MF-induced membrane depolarization to the MF flux density. We used the following values to estimated the threshold values: *R* = 0.15 m, λ = 10^−3^ m, and *f* = 60 Hz. The MF flux density value as a function of the MF-induced membrane depolarization *dV*, depending on different τ values, is shown in Figure 7.

We have shown based on our statistical analysis that a significant EEG alpha power modulation occurs in the model for a *dV* value between 250 and 500 μV. Given the uncertainty on the polarization time constant, it is only possible to provide an estimate of the MF flux density threshold at 60 Hz which should result in a significant decrease of EEG alpha activity. Assuming an intermediate *dV* value as a threshold value (375 μV), the corresponding threshold MF flux density would range between 15 and 75 mT, for polarization time constants between 1 and 15 ms. Let us also mention that this threshold value is, as shown above, depending on the input noise level. Uncertainties on the values of the polarization time constant and on the input noise level are problematic to estimate a more precise MF flux density threshold value. Depending on the neural elements activated by the induced electric field, this polarization time constant can be very different (higher for the whole soma than for fibers for example). For a neuron soma, the polarization time constant would be of several milliseconds; whereas if the membrane depolarization occurs at the level of Ranvier nodes, than the polarization time constant is considerably smaller, around 20 μs (Gianni et al., 2006). Therefore, it is reasonable to assume that the polarization time constant is in the low millisecond range. As an example, a polarization time constant between 1 and 5 ms would result in a threshold value between 15 and 25 mT for a 60 Hz MF. The validation of these values will require experimental recordings performed in humans, which will be performed in the near future in our group (Legros et al., 2012a,b).

## Discussion

In this paper, we have developed an innovative application of neural mass models, i.e., the study of how extremely low-frequency MF such as power-line MF interact with brain activity. Indeed, this is the first time that this problem is tackled using neural mass modeling. We have shown that 60 Hz MF exposure can result in a modulation of the EEG alpha rhythm, even for small membrane depolarization values (<1 mV). For reasonable polarization time constant values, the model predicts that 60 Hz MF between 15 and 25 mT could induce a systematic decrease in EEG alpha power. Furthermore, the neural mass model that we have developed includes, in a simplified manner, a contribution of synaptic plasticity processes. To our knowledge, this is the first attempt to include a contribution of calcium-related processes into changes of effective connectivity in neural mass models. The contribution of calcium currents on synaptic weights changes is obviously overly simplified in our model since the calcium concentration is modeled as a low-pass filtered version of the EEG. Nevertheless, it represents a first step that could serve as a basis in future models integrating more biophysically detailed models of synaptic plasticity. Let us mention that Robinson (2011) proposed a neural field model including synaptic plasticity, though in a different manner. Indeed, this model used the relative phase between pre- and post-synaptic neural populations to compute the synaptic weight changes due to an STDP rule. In the present paper, we have intended to provide the bases for mechanism-based neural mass models, on the grounds of a reliable synaptic plasticity model (Shouval et al., 2002a,b). Expanding our model using modeling principles of Robinson (2011) could be a solution so include the effect of spike timing perturbation induced by 60 Hz MF on neural mass activity.

One hypothesis investigated was that changes in post-synaptic calcium concentration could modulate synaptic weights, resulting in a lasting modulation of brain tissue dynamics. Obviously, our model of synaptic plasticity is still simplified and does not explicitly model the voltage-dependence of calcium currents through NMDA receptors. From our results, it appears that, despite a modulation in post-synaptic calcium concentration taking some time to build up, and lasting several minutes after the exposure, these changes are too small to impact neural mass dynamics. However, there is another important mechanism by which receptor trafficking and synaptic plasticity could be modulated by 60 Hz MF exposure. There is indeed a convergence of theoretical (Reato et al., 2010; Stodilka et al., 2011) and experimental (Radman et al., 2007) studies that illustrate the possibility for weak membrane depolarizations induced by electric fields to impact spike timing. Indeed, Radman et al. (2007) have shown that, due to the non-linear properties of neuron membranes, small membrane depolarizations can modulate spike timing. Since post-synaptic calcium currents play the role of “coincidence detector” between pre- and post-synaptic spikes, a perturbation of spike timing could impact receptor trafficking and synaptic weight changes. The challenge is to consider these mechanisms in neural mass or neural field models, which are rate-coding based and not time-coding based. A recent study proposed how to consider plasticity rules based on spike timing in neural field theory (Robinson, 2011), providing a possibility to investigate the impact of 60 Hz MF perturbation on spike timing at a mesoscopic scale. The perturbation of spike timing by 60 Hz MF exposure will be considered in a future extension of the model presented in this paper, since this synaptic plasticity pathway could be more prone to small membrane perturbations due to the 60 Hz MF exposure, and could induce lasting effects in neural dynamics.

Using our model, we have also investigated the effect of the small 60 Hz membrane depolarization induced by 60 Hz MF exposure depending of the input noise level of the model. Our suggests that the threshold value in MF flux density for which significant changes can be detected in the EEG alpha frequency band is a function of the model input noise level. More specifically, for lower values of input noise level, the decrease in EEG alpha power is higher than for higher input noise level values. However, these results do not imply that stochastic resonance effects are present, which could be tested however by testing many different values of *dV*, and identify a range of *dV* values for which the modulation of EEG alpha power would be present. The stochastic resonance mechanism has been already explored in the literature to explain the effects of 60 Hz MF exposure, and constitutes a possibility of future study using our model. This result on the importance of the input noise level has also important implications for the detection of EEG alpha power modulation in humans due to 60 Hz MF exposure. We predict that the threshold in MF flux density at 60 Hz, required to modulate systematically the EEG in the occipital cortex, is lower a condition when the ambient light is low, compared to the effects of the same exposure using a high ambient light level. Indeed, EEG alpha oscillations decrease in the occipital cortex in humans due to the higher input noise level. Therefore, it might be relevant to study a variation of 60 Hz MF threshold values in humans at the level of the occipital cortex using different intensities of ambient light. If such experimental evidence was provided, that would be a precious piece of information that could be of interest to agencies such as ICNIRP. Another indication that the prediction that the level of input is important in the physiological outcome is that the perception of magnetophosphenes and the threshold at which they can be observed is light intensity-dependent (Lövsund et al., 1980). This motivates a future human experimental study using in parallel our neural mass model in order to provide an improved knowledge on the underlying interaction mechanisms. Another valuable insight from the model is the differential effect observed on EEG alpha activity depending on the neuronal populations modulated (pyramidal neurons only, or pyramidal neurons and slow/fast inhibitory interneurons). Since the model predicts a different outcome in the case where slow inhibitory interneurons are also modulated (smaller decrease in alpha activity), this offers a possibility to discriminate in future EEG data acquired in humans which neuronal populations are modulated by the MF exposure. This adds further support for the use of neural mass models to study the effects of power-line MF on human brain activity, since they can offer a deeper insight into the experimental data in order to clarify the interaction mechanisms involved.

Among the limitations of our approach, let us mention first the absence of ephactic interactions. It has been indeed demonstrated that post-synaptic potentials can induce in neighboring cells a small but measurable polarization (for a review, see Weiss and Faber, 2010). In the cortex, where the axons of pyramidal axons have a similar and consistent orientation, it is likely that ephactic interactions could enhance the effect of weak membrane depolarizations. Therefore, the presence of ephactic interactions should lower the threshold for detectable modulations of neuronal activity, and should be included in future biophysical models studying the effects of low-frequency MF on cortical activity. Second, our model of synaptic plasticity is a significant simplification compared to the biophysically detailed model by Shouval et al. (2002a,b). The present model could be extended by including a more detailed model of the detailed processes underlying receptor trafficking at the synaptic level. Third, the exact orientation of pyramidal axons with respect to the induced electric field was not taken into account, since it was assumed that the orientation was “ideal” (electric field parallel to pyramidal neuron axons). Fourth, we assumed that the power-line MF interacts with brain tissue *via* the induced electric field. However, there is evidence that the MF itself could interact with cellular signaling, and induce biological effects (Pilla, 2012). Studying such phenomena appears however out of reach with our proposed model. Let us note that the possibility that either the MF or the induced electric field modulate neuronal activity is not exclusive, and both mechanisms might even turn out to be complementary and have effects on different cellular components.

Finally, let us mention that, in most studies investigating the effects of low-frequency MF on the human EEG, the data analyzed is from before or after exposure, not during. There is indeed an experimental difficulty in recording the EEG during exposure to low-frequency MF. However, it is possible to compensate using specific signal processing techniques, such as wavelet-based methods (Modolo et al., 2011). We believe that such signal processing techniques applied to EEG acquired during 60 Hz MF exposure, combined with the neural mass model proposed in this paper, could provide an integrated framework for a thorough understanding of power-line frequency MF on human brain activity.

## Concluding remarks

We have presented a novel application of neural field models, in the context of brain exposure to 60 Hz MF. The model takes into account different neural populations in cortical tissue, synaptic kinetics proper to each type of synapse considered, synaptic connectivity patterns inspired from neuroanatomy, and a simplified model of synaptic plasticity based on the “calcium-control” hypothesis. The model includes the interaction with the electric field induced by 60 Hz MF exposure, and results in a time-varying membrane depolarization. Using this model, we have shown that membrane depolarization between 250 and 500 μV at 60 Hz is sufficient to induce a significant decrease in EEG alpha power. We also conclude that the modulation of post-synaptic calcium currents by 60 Hz MF exposure does not appear to predict the lasting effects observed experimentally. Future work should investigate the role of spike timing perturbation by the induced electric field during 60 Hz MF exposure, which is another candidate mechanism to induce plastic changes and lasting changes in neuronal activity. The models provides predictions that can, and will be, tested in experimental protocols during which humans will be exposed to increasing levels of 60 Hz MF exposure up to 50 mT (Legros et al., 2012a,b). Thorough comparison of experimental data with model predictions will constitute a unique opportunity for the validation and calibration of this neural mass model, which might become a relevant tool in the assessment of public and workers exposure to environmental MF, and assisting in the development and evaluation of guidelines developed by ICNIRP.

### Conflict of interest statement

The authors declare that the research was conducted in the absence of any commercial or financial relationships that could be construed as a potential conflict of interest.

## Figures and Tables

**Table A1:**
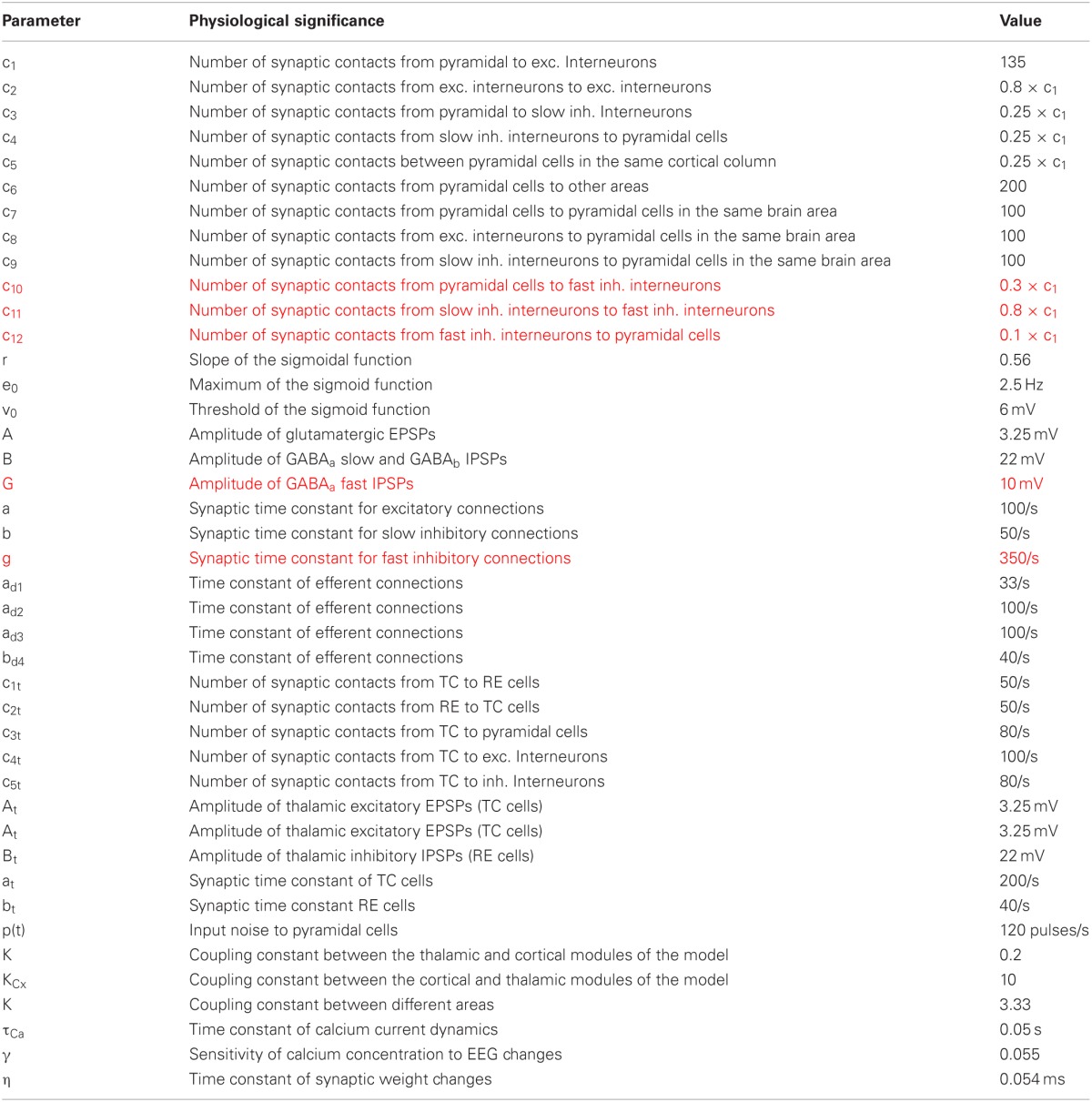
**Summary of the parameters used in the model with their physiological meaning and their values**.

Novel parameters compared to Sotero and Trujillo-Barreto (2008) are highlighted in red.

## References

[B1] Amari S. (1977). Dynamics of pattern formation in lateral-inhibition type neural fields. Biol. Cybern. 27, 77–87 911931 10.1007/BF00337259

[B2] Bikson N. Inoue M. Akiyama H. Deans J. K. Fox J. E. Miyakawa H. (2004). Effects of uniform extracellular DC electric fields on excitability in rat hippocampal slices *in vitro*. J. Physiol. 557(Pt 1), 175–190 10.1113/jphysiol.2003.055772 14978199 PMC1665051

[B3] Bojak I. Oostendorp T. F. Reid A. T. Kotter R. (2010). Connecting mean field models of neural activity to EEG and fMRI data. Brain Topogr. 23, 139–149 10.1007/s10548-010-0140-3 20364434

[B4] Collingridge G. L. Isaac J. T. Wang Y. T. (2004). Receptor trafficking and synaptic plasticity. Nat. Rev. Neurosci. 5, 952–962 10.1038/nrn1556 15550950

[B5] Cook C. M. Thomas A. W. Keenliside L. Prato F. S. (2005). Resting EEG effects during exposure to a pulsed ELF magnetic field. Bioelectromagnetics 26, 367–376 10.1002/bem.20113 15887255

[B6] Cook C. M. Thomas A. W. Prato F. S. (2004). Resting EEG is affected by exposure to a pulsed ELF magnetic field. Bioelectromagnetics 25, 196–203 10.1002/bem.10188 15042628

[B7] Corbacio M. Brown S. Dubois S. Goulet D. Prato F. S. Thomas A. W. (2011). Human cognitive performance in a 3 mT power-line magnetic field. Bioelectromagnetics 23, 620–633 10.1002/bem.20676 21544842

[B8] Crasson M. (2003). 50-60 Hz electric and magnetic field effects on cognitive function in humans: a review. Radiat. Prot. Dosimetry 106, 333–340 14690276 10.1093/oxfordjournals.rpd.a006369

[B9] Deans J. K. Powell A. D. Jefferys J. G. (2007). Sensitivity of coherent oscillations in rat hippocampus to AC electric fields. J. Physiol. 583(Pt 2), 555–565 10.1113/jphysiol.2007.137711 17599962 PMC2277040

[B10] Fröhlich F. McCormick D. A. (2010). Endogenous electric fields may guide neocortical network activity. Neuron 67, 129–143 10.1016/j.neuron.2010.06.005 20624597 PMC3139922

[B11] Ghione S. Seppia C. D. Mezzalma L. Bonfglio L. (2005). Effects of 50 Hz electromagnetic fields on electroencephalography alpha activity, dental pain threshold and cardiovascular parameters in humans. Neurosci. Lett. 382, 112–117 10.1016/j.neulet.2005.02.072 15911132

[B12] Gianni M. Liberti M. Appollonio F. D'Inzeo G. (2006). Modeling electromagnetic fields detectability in a HH-like neuronal system: stochastic resonance and window behavior. Biol. Cybern. 94, 118–127 10.1007/s00422-005-0029-5 16369796

[B12a] Grimbert F. Faugeras O. (2006). Bifurcation analysis of Jansen's neural mass model. Neural Comput. 18, 3052–3068 10.1162/neco.2006.18.12.3052 17052158

[B13] ICNIRP—International Commission on Non-Ionizing Radiation Protection. (2010). Guidelines for Limiting Exposure to Time-Varying Electric and Magnetic Fields (1 Hz – 100 kHz). Health Phys. 99, 818–836 10.1097/HP.0b013e3181f06c86 21068601

[B14] Jansen B. H. Rit V. G. (1995). Electroencephalogram and visual evoked potential generation in a mathematical model of coupled cortical columns. Biol. Cybern. 73, 357–366 7578475 10.1007/BF00199471

[B15] Jefferys J. G. Deans J. Bikson M. Fox J. (2003). Effects of weak electric fields on the activity of neurons and neuronal networks. Radiat. Prot. Dosimetry 106, 321–323 14690274 10.1093/oxfordjournals.rpd.a006367

[B17] Legros A. Beuter A. (2005). Effect of a low intensity magnetic field on human motor behavior. Bioelectromagnetics 26, 657–669 10.1002/bem.20161 16189826

[B18] Legros A. Corbacio M. Beuter A. Modolo J. Goulet D. Prato F. S. (2012a). Neurophysiological and behavioral effects of a 60 Hz, 1800 microtesla magnetic field in humans. Eur. J. Appl. Physiol. 112, 1751–1762 10.1007/s00421-011-2130-x 21894451

[B19] Legros A. Modolo J. Goulet D. Plante M. Souques M. Deschamps F. (2012b). Threshold for a systematic neurophysiological response to 50 and 60 Hz magnetic fields up to 50 millitesla, 34th Annual Conference of the Bioelectromagnetics Society (Brisbane, QLD).

[B20] Legros A. Miller J. E. Modolo J. Corbacio M. Robertson J. R. Goulet D. (2010) Is finger tapping induced brain activation modulated by an exposure to a 60 Hz, 3000 μT magnetic field? in 32nd Annual Conference of the Bioelectromagnetics Society (Seoul).

[B21] Legros A. Miller J. Modolo J. Corbacio M. Robertson J. Goulet D. (2011). Multi-modalities investigation of 60 Hz magnetic field effects on the human central nervous system. Electra 256, 14–18

[B22] Lövsund P. Oberg P. A. Nilsson S. E. Reuter T. (1980). Magnetophosphenes: a quantitative analysis of thresholds. Med. Biol. Eng. Comput. 18, 326–334 6968384 10.1007/BF02443387

[B23] Miller J. E. Modolo J. Robertson J. R. Corbacio M. Dubois S. Goulet D. (2010). Effects of a 60 Hz magnetic field exposure on human brain activity during a mental rotation task as measured by fMRI, 32nd Annual Conference of the Bioelectromagnetics Society (Seoul).

[B24] Modolo J. Bhattacharya B. Edwards R. Campagnaud J. Legros A. Beuter A. (2010). Using a virtual cortical modulate implementing a neural field model to modulate brain rhythms in Parkinson's disease. Front. Neurosci. 4:45 10.3389/fnins.2010.00045 20730081 PMC2920509

[B25] Modolo J. Juen N. Robertson J. A. Thomas A. W. Legros A. (2011). EEG frequency analysis of 60 Hz magnetic field exposure within the MRI, Conference of the Conseil International des Grands Réseaux Electriques (CIGRE) (Paris).

[B26] Molaee-Ardekani B. Benquet P. Bartolomei F. Wendling F. (2010). Computational modeling of high-frequency oscillations at the onset of neocortical partial seizures: from “altered structure” to “dysfunction”. Neuroimage 52, 1109–1122 10.1016/j.neuroimage.2009.12.049 20034581

[B27] Molaee-Ardekani B. Márquez-Ruiz J. Merlet I. Leal-Campagnario R. Gruart A. Sánchez-Campusano R. (2013). Effects of transcranial direct current stimulation (tDCS) on cortical activity: a computational modeling study. Brain Stimul. 6, 25–39 10.1016/j.brs.2011.12.006 22420944

[B28] National Institute of Environmental Health Sciences of the National Institutes of Health. (1998). Assessment of Health Effects From Exposure to Power-Line Frequency Electric and Magnetic Fields, eds Protier C. J. Wolfe M. S. (Research Triangle Park, NC: NIH publication No. 98–3981).

[B29] Pilla A. (2012). Electromagnetic fields instantaneously modulate nitric oxide signaling in challenged biological systems. Biochem. Biophys. Res. Commun. 426, 330–333 10.1016/j.bbrc.2012.08.078 22940137

[B30] Radman T. Ramos R. L. Brumberg J. C. Bikson M. (2009). Role of cortical cell type and morphology in subthreshold and suprathreshold uniform electric field stimulation *in vitro*. Brain Stimul. 2, 215–228 10.1016/j.brs.2009.03.007 20161507 PMC2797131

[B31] Radman T. Su Y. An J. H. Parra L. C. Bikson M. (2007). Spike timing amplifies the effects of electric fields on neurons: implications for endogenous field effects. J. Neurosci. 27, 3030–3036 10.1523/JNEUROSCI.0095-07.2007 17360926 PMC6672570

[B32] Reato D. Rahman A. Bikson M. Parra L. C. (2010). Low-intensity electrical stimulation affects network dynamics by modulating population rate and spike timing. J. Neurosci. 30, 15067–15079 10.1523/JNEUROSCI.2059-10.2010 21068312 PMC3500391

[B33] Robinson P. A. (2011). Neural field theory of synaptic plasticity. J. Theor. Biol. 285, 156–163 10.1016/j.jtbi.2011.06.023 21767551

[B34] Shouval H. Z. Castellani G. C. Blais B. S. Yeung L. C. Cooper L. N. (2002a). Converging evidence for a simplified biophysical model of synaptic plasticity. Biol. Cybern. 87, 383–391 10.1007/s00422-002-0362-x 12461628

[B35] Shouval H. Z. Bear M. F. Cooper L. N. (2002b). A unified model of NMDA receptor-dependent bidirectional synaptic plasticity. Proc. Natl. Acad. Sci. U.S.A. 99, 10831–10836 10.1073/pnas.152343099 12136127 PMC125058

[B35a] Stodilka R. Z. Modolo J. Prato F. S. Robertson J. A. Cook C. Patrick J. (2011). Pulsed magnetic field exposure induces lasting changes in neural network dynamics. Neurocomputing 74, 2164–2175

[B37] Sotero R. C. Trujillo-Barreto N. J. (2008). Biophysical model for integrating neuronal activity, EEG, fMRI and metabolism. Neuroimage 39, 290–309 10.1016/j.neuroimage.2007.08.001 17919931

[B39] Weiss S. A. Faber D. S. (2010). Field effects in the CNS play functional roles. Front. Neural Circuits 4:15 10.3389/fncir.2010.00015 20508749 PMC2876880

[B40] Wendling F. Bartolomei F. Bellanger J. J. Chauvel P. (2002). Epileptic fast activity can be explained by a model of impaired GABAergic dendritic inhibition. Eur. J. Neurosci. 15, 1499–1508 10.1046/j.1460-9568.2002.01985.x 12028360

[B41] Wendling F. Hernandez A. Bellanger J. J. Chauvel P. Bartolomei F. (2005). Interictal to ictal transition in human temporal lobe epilepsy: insights from a computational model of intracerebral EEG. J. Clin. Neurophysiol. 22, 343–356 16357638 PMC2443706

[B42] Wilson H. R. Cowan J. D. (1973). A mathematical theory of the functional dynamics of cortical and thalamic nervous tissue. Kybernetik 13, 55–80 4767470 10.1007/BF00288786

